# Supplementary material for: Cross continental increase in methane ebullition under climate change
Source: Nat Commun. 2017 Nov 22;8:1682. doi: 10.1038/s41467-017-01535-y (PMC5700168; doi:10.1038/s41467-017-01535-y)
Supplement: Supplementary file 1 — Supplementary Information [file 41467_2017_1535_MOESM1_ESM.pdf]

Supplementary Table 1 | Characteristics of freshwater ecosystems in Asia, North America, and Europe used to model the relationships between temperature and CH_4 ebullition (Table 1). Concentrations of compounds apply to surface water.

System	Mean ME[b] (mg m^{-2} d^{-1})	Depth (m)	# of water bodies	Sedimentation (m yr^{-1})	ChL *a* (µg L^{-1})	OC (mg L^{-1})	TN (mg L^{-1})	TP (µg L^{-1})	NH_4^+-N (mg L^{-1})	NO_3^--N (mg L^{-1})	PO_4^{3-}-P (µg L^{-1})	Latitude	Longitude	Reference
Subtropical eutrophic city pond (D1)	744	4	1	-[c]	-	-	5.8 – 6.1	280 – 510	0.47 – 2.49	0.3 – 2.3	-	32° 02'N	118° 52'E	1
Subtropical eutrophic city pond (D2)	953	3.5	1	-[c]	-	-	5.8 – 6.1	280 – 510	0.47 – 2.49	0.3 – 2.3	-	32° 02'N	118° 52'E	1
Subtropical eutrophic city pond (D3)[a]	1309	2.8	1	-[c]	-	-	5.8 – 6.1	280 – 510	0.47 – 2.49	0.3 – 2.3	-	32° 02'N	118° 52'E	1
Post-glacial lakes[a]	59	< 1 - 7	3	-	-	10[d]	-	-	-	-	-	68° 21'N	19° 03'E	2,3
Temperate river Saar (ABT1)	3015	4	1	0.29	-	-	-	-	0.10 – 0.49	1.9 – 3.8	93 – 341	49° 34'N	6° 37'E	4
Temperate river Saar (ABT2)	1158	2	1	0.07	-	-	-	-	0.10 – 0.49	1.9 – 3.8	93 – 341	49° 34'N	6° 36'E	4
Temperate river Saar (ABT3)	1813	2.7	1	0.10	-	-	-	-	0.10 – 0.49	1.9 – 3.8	93 – 341	49° 34'N	6° 36'E	4
Temperate river Saar (ABT4)[a]	1270	2.2	1	0.15	-	-	-	-	0.10 – 0.49	1.9 – 3.8	93 – 341	49° 30'N	6° 34'E	4
Boreal meso-eutrophic forest ponds[a]	141	0.6 - 0.9	10	-	0.7 – 6.4	8.1 – 18.2[d]	0.2 – 0.6	14 – 48	-	-	-	48° 23'N	71° 25'W	5
Temperate eutrophic city pond[a]	625	< 1 - 2	1	-	36.5 – 227.6	7.5 – 18.2[e]	1.3 – 3.3	94 – 203	0.03 – 1.27	0 – 0.9	27 – 91	51° 47'N	5° 51'E	Unpublished
Temperate farm ponds[a]	372	1	2	-	-	-	-	-	-	-	-	42° 15'N	84° 03'W	6
Mesocosm experiment[a]	84	1.35	8[f]	-	0 – 56.0	2.4 – 6.4[d]	0.3 – 1.4	3 – 59	0 – 1.97	0 – 1.4	0 – 127	51° 59'N	5° 40'E	This study

[a]Systems presented in Fig. 1.
[b]Mean methane ebullition (ME) at 20°C.
[c]Different water depths of sublocations (D1-D3) are mainly the consequence of differences in sedimentation rates.
[d]Dissolved organic carbon.
[e]Total organic carbon.
[f]Number of mesocosms.

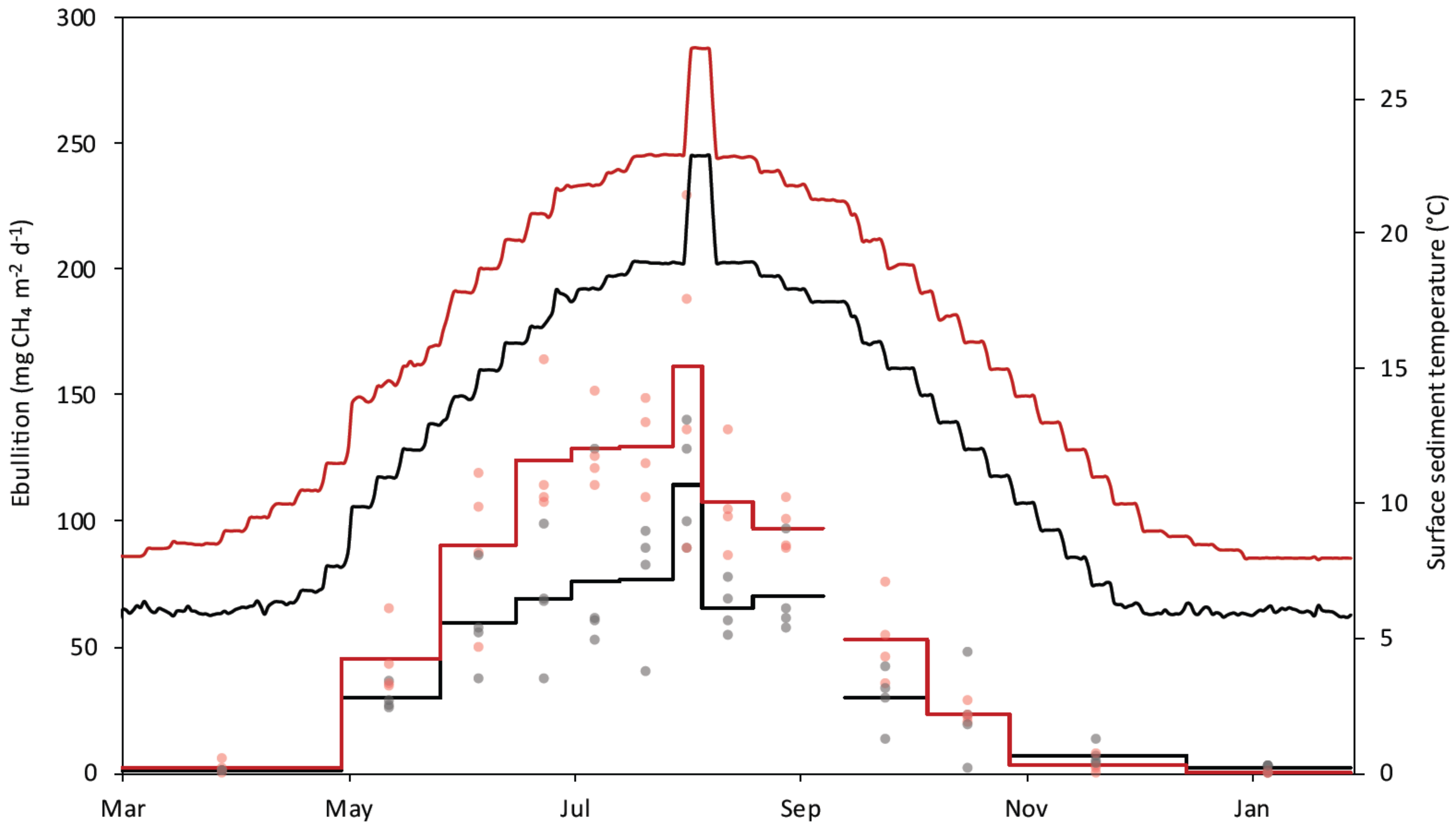

Supplementary Figure 1 | Surface sediment temperature & mean CH_4 ebullition for the control and warm treatment (+4°C). Upper lines denote surface sediment temperature, whereas lower lines denote mean CH_4 ebullition for the control (black) and warm (red) treatment, respectively. From 3 till 10 August, a heat wave (+4°C) was applied to both treatments.

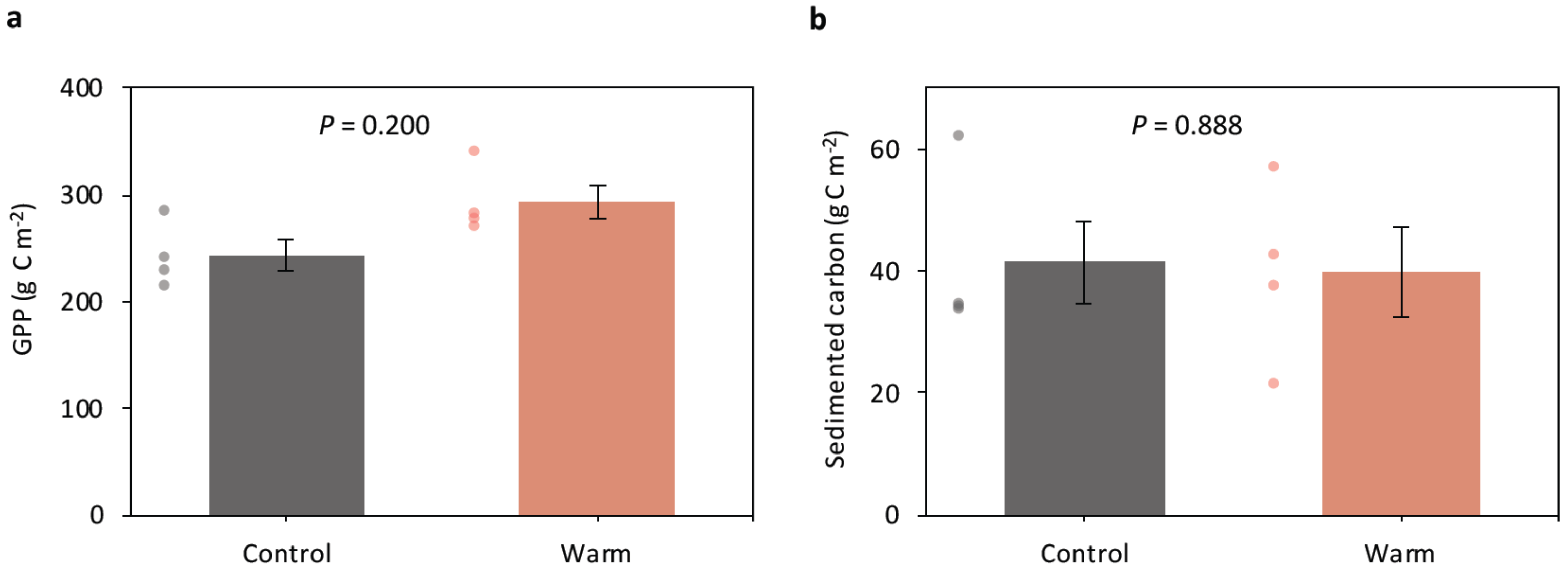

Supplementary Figure 2 | Cumulative annual Gross Primary Production (GPP) (a) and sedimented carbon (C) (b) for the control and warm treatment. Error bars denote 1 standard error of the mean. (n = 4). Differences between treatments were tested with a Mann-Whitney and t-test, respectively.

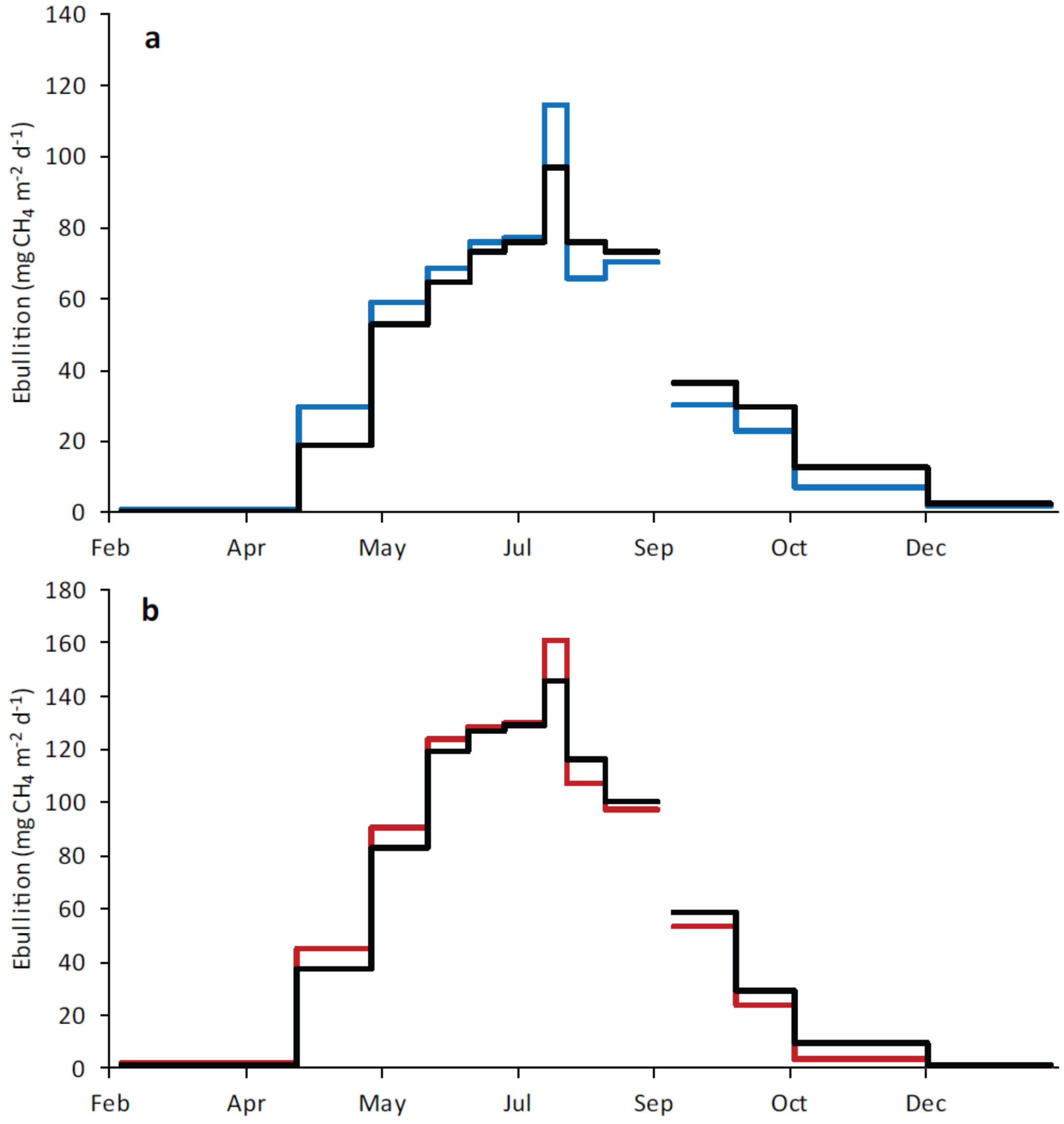

Supplementary Figure 3 | Effect of changing CH_4 solubility on CH_4 ebullition throughout the year for the control (a) and warm treatment (b). Colored line denotes the measured ebullitive flux, black line denotes calculated ebullitive flux assuming no changes in CH_4 solubility in sediment pore water.

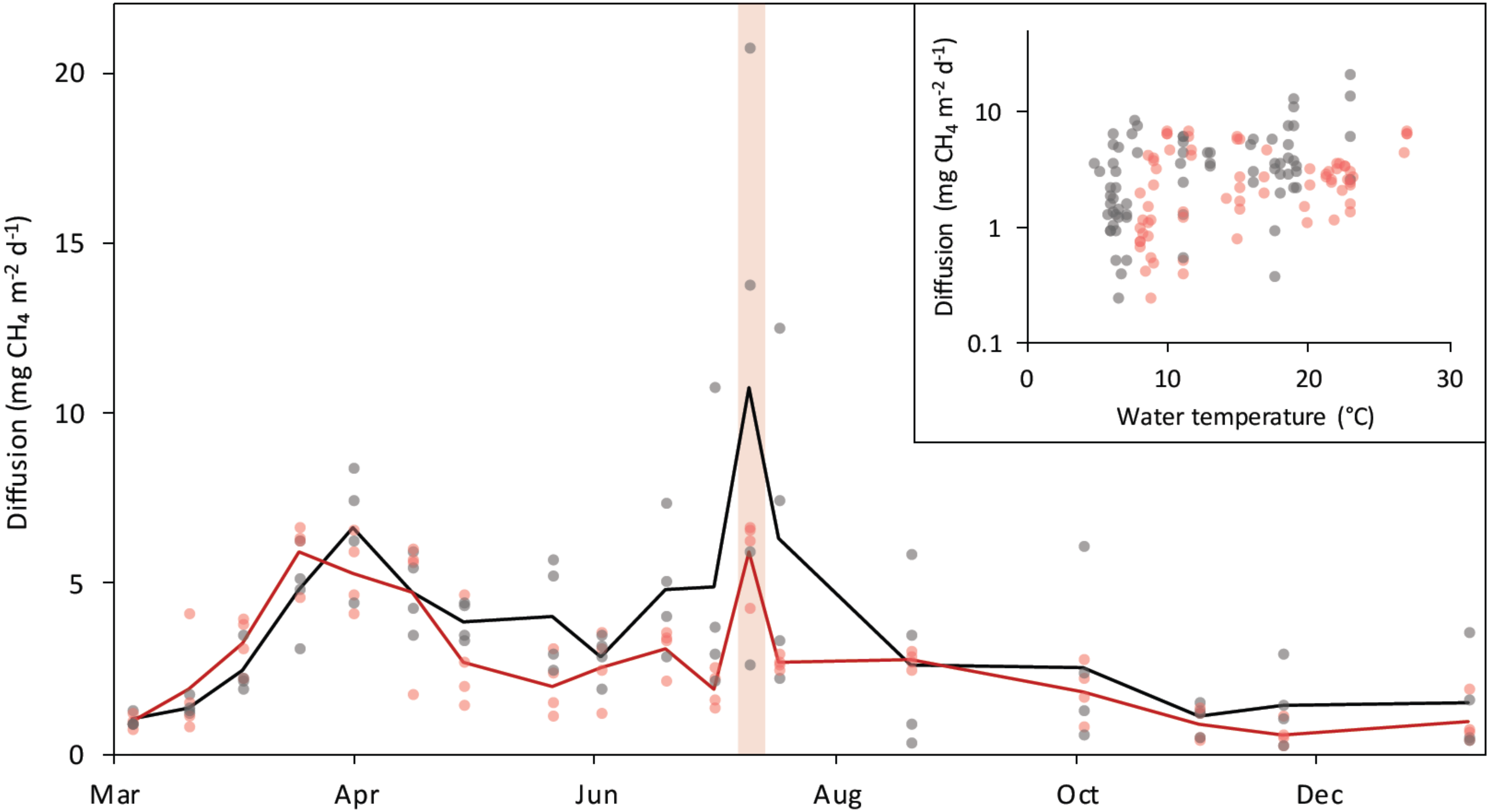

Supplementary Figure 4 | Mean diffusive CH_4 emissions for the control and the warm (+4°C) mesocosms. Red circles and lines (arithmetic mean) denote warm treatment, black ones denote control. From 3 till 10 August, a heat wave (+4°C) was applied to both treatments (red shading). Top right scatter plot shows correlation between temperature and diffusive CH_4 emission for the control and warm treatment (in both cases Spearman rho = 0.40; n = 72; $P < 0.001$).

Supplementary References

1 Gao, Y. *et al.* Estimation of N_2 and N_2O ebullition from eutrophic water using an improved bubble trap device. *Ecological Engineering* **57**, 403-412 (2013).

2 Wik, M. *et al.* Energy input is primary controller of methane bubbling in subarctic lakes. *Geophysical Research Letters* **41**, 555-560 (2014).

3 Wik, M. Emission of methane from northern lakes and ponds. PhD dissertation. Stockholm University, Department of Geological Sciences (2016).

4 Wilkinson, J., Maeck, A., Alshboul, Z. & Lorke, A. Continuous seasonal river ebullition measurements linked to sediment methane formation. *Environmental Science & Technology* **49**, 13121-13129 (2015).

5 DelSontro, T., Boutet, L., St-Pierre, A., del Giorgio, P. A. & Prairie, Y. T. Methane ebullition and diffusion from northern ponds and lakes regulated by the interaction between temperature and system productivity. *Limnology and Oceanography* **61**, S62-S77 (2016).

6 Baker-Blocker, A., Donahue, T. M. & Mancy, K. H. Methane flux from wetlands areas. *Tellus* **29**, 245-250 (1977).
